# Outpatient visits after retirement in Europe and the US

**DOI:** 10.1007/s10754-016-9191-7

**Published:** 2016-06-30

**Authors:** Anikó Bíró

**Affiliations:** Department of Health Economics, Corvinus University of Budapest, Fővám tér 8, Budapest, 1093 Hungary

**Keywords:** Health care demand, Outpatient care, Retirement, Gatekeeping, I10, I12, J14

## Abstract

I conduct an empirical analysis of the relation between retirement and outpatient care use in Europe and the US, and investigate the potential driving factors of that. I link the empirical analysis to a theoretical model of medical care demand. I document that pensioners tend to visit a doctor with higher probability and more often than the rest of the 50+ population. Ceteris paribus, being retired implies 3–10 % more outpatient visits in Europe. The estimates are of similar magnitude in the US. The paper contributes to the understanding of how population ageing plays a part in the rising health care expenditures. I find evidence that retirement related individual characteristics, increasing leisure time and stronger health preferences all contribute to the positive relation between retirement and outpatient care use, which is mainly driven by the healthier individuals. The gatekeeper role of general practitioners can mitigate the increased demand for outpatient care services after retirement.

## Introduction

Expenditures on outpatient care make up around 33 % of total health expenditures in the OECD. Spending on outpatient care is increasing, between 2009 and 2013 its real annual growth rate was 1.7 %, only the long-term care expenditures had a higher growth rate (OECD 2015). This paper has two aims. First, to analyse and provide explanations to the relations between retirement and outpatient care use, both theoretically and empirically. Second, to investigate some of the cross country differences in the relation between retirement and outpatient care use. This analysis can help us better understand the determinants of the demand for outpatient care, which issue is relevant for policies aiming at reducing public health expenditures while improving population health.

Based on data from the Survey of Health, Ageing and Retirement in Europe (SHARE) and the Health and Retirement Study (HRS), pensioners are more likely to use outpatient care than the rest of the population of comparable age. This can be observed both in Europe and the US, and even if age is controlled for. Also, within Europe, pensioners on average visit a specialist more frequently than those who are working. I analyse how age, socioeconomic conditions, health, health preferences and time constraints can contribute to the explanation of the retirement—outpatient care use gradient. The baseline results are based on pooled non-linear regression models, which are then extended with interaction terms between retirement status and indicators of health, time constraints and health preferences. I also analyse the relation between retirement and outpatient care use by countries and by country groups according to institutional characteristics. Endogeneity issues are addressed with using official retirement ages and observed population level retirement rates as instruments. The empirical analysis corresponds to the theoretical framework of utility maximisation.

Bonsang et al. (2012), Coe and Zamarro (2011), and Neuman (2008), among others, use either of the same data sets to analyse the health effects of retirement (finding positive effect on physical health and negative effect on cognitive functioning), without focusing on the relationship between retirement and outpatient care use. Although there is a rich literature on the health effects of retirement (e.g. two recent studies from Europe are Eibich (2015) and Hallberg et al. (2015), finding positive health effects and decreased health care use if health is *not* controlled for), my paper differs from that literature in focusing on the effects of retirement on outpatient care use, *conditional on observed health*.

This paper is related to two strands of the literature: a more general strand that explains the demand for medical care, and a more specific strand that documents the utilisation of health care in relation to retirement. The utility maximisation framework that I apply fits into the line of literature originating from the seminal work of Grossman (1972) who derives a basic model of optimal stock of health capital and hence of investment in health. Acton (1975) focuses on how nonmonetary factors affect the demand for medical care. In my paper the focus is also mainly on nonmonetary determinants of outpatient care use. Other papers such as Cameron et al. (1988) analyse both theoretically and empirically the demand for health care, without focusing on the effect of retirement.

There is a lack of recent literature that would investigate and explain the relationship between health care use and retirement. Some earlier papers find mixed results on this relation mainly based on US data. There is even less evidence on the relation within Europe. Also, the existing papers are mostly of descriptive nature. The results of Soghikian et al. (1991), based on data from Northern California, do not support a positive relationship between leisure time and health care use by retirees. Similarly, based on Canadian data, Shapiro and Roos (1982) find that retirement does not necessarily imply higher utilization of ambulatory care. On the other hand, based on US data, Boaz and Muller (1989) find that retirees are significantly more likely to use outpatient care services than the self-employed, and their expected number of doctoral visits is also higher. However, they do not find such significant differences between the employees and pensioners. I find more robust albeit relatively small differences between the pensioners and the rest of the 50+ population in terms of outpatient care use. The differences compared to the earlier literature can stem from several factors: later time periods are covered, different data sets are used, covering different countries, and there are differences in the empirical strategy as well.

Overall, I find evidence for a positive relation between retirement and outpatient care use, both in Europe and the US. These results are conditional on health and other individual characteristics, thus do not contradict the results in the literature on the health improving effects of retirement but suggest that ceteris paribus, the demand for outpatient care increases after retirement. Focusing on the European data, further specification checks reveal that the positive relations are driven by the healthier individuals, those who were more time constrained while working, and those for whom health seems to matter more. Cross country comparisons do not indicate that the retirement—outpatient care use gradient would clearly be related to outpatient care co-payments. However, the results suggest that the gatekeeping role of GPs can reduce the retirement induced demand for outpatient care.

## Model

In this section I present a simple model of health care use that can serve as a framework for the empirical analysis and discussion. My aim is to present a model which has a closed form solution, but can capture well the relationship between retirement and health care use. The model’s solution is derived in the Appendix. The model follows Grossman (1972) in the sense that medical care can increase the stock of health capital, which has a positive effect on utility. For the sake of simplicity, it is a static health care demand model with Cobb-Douglas utility of consumption (*C*) and health (*H*) with parameter $0<\gamma <1$, and where health is a linear function of health care use (*M*). Health is also allowed to depend on retirement status, with $\rho$ being the binary indicator which equals one if someone is retired and zero if working.^1^ In this simple specification, the effect of ageing on health is captured by the effect of retirement on health. $\phi _{0}$ is the health level without health care use, which is at the same time the lowest possible level of health (conditional on retirement status).

1 $$\begin{aligned} \max _{C\ge 0,M\ge 0}U= & {} C^{\gamma }H^{1-\gamma } \end{aligned}$$

2 $$\begin{aligned} H= & {} \phi _{0}\left( \rho \right) +\phi _{1}M;\, \phi _{0},\phi _{1}\ge 0. \end{aligned}$$

The budget constraint is:

3 $$\begin{aligned} C+pM=\rho R+(1-\rho )w\left( W_{0}+W_{1}H-tM\right) , \end{aligned}$$

where the consumption goods have unit price, *p* is the price of medical care, *R* is pension income, *w* is the hourly wage, $W_{0}$ is the working hours if someone has health asset $H=0$, $W_{1}$ is the increase in working hours due to an unit of improvement in health, and *t* is the time cost of health care use. To keep the model as simple as possible, I assume that retirement status, income, working hours conditional on health and hourly wages are all exogenous. Although retirement status is likely to depend on health, my aim is not to model the retirement decision but to analyse the health care use differences between the retired and working individuals, therefore it is sufficient to capture the retirement-health relations with allowing health to depend on retirement status and then solve the model for the retired and the working. I return to the issue of endogenous retirement in relation to the empirical strategy in “Instrumental variables” section.

Solving the model yields that for the retired ($\rho =1$) the optimal demand for medical care is:

4 $$\begin{aligned} M_{\rho =1}=-\gamma \frac{\phi _{0}\left( \rho =1\right) }{\phi _{1}}+\frac{ \left( 1-\gamma \right) R}{p}. \end{aligned}$$

For the working population ($\rho =0$) the optimal demand is:

5 $$\begin{aligned} M_{\rho =0}=-\gamma \frac{\phi _{0}\left( \rho =0\right) }{\phi _{1}}+\frac{ \left( 1-\gamma \right) w\left[ W_{0}+W_{1}\phi _{0}\left( \rho =0\right) \right] }{p-w\left( W_{1}\phi _{1}-t\right) }. \end{aligned}$$

Based on this model, there are multiple possible reasons why health care demand might increase after retirement ($M_{\rho =1}>M_{\rho =0}$). First, it can be the result of decreasing out-of-pocket cost of medical care (*p*). Second, changing health preferences (decreasing $\gamma$) can also provide explanation. Decreasing time costs (*t*) can also play a role—in the above model I assumed that the cost of time is zero after retirement. If the marginal health benefit of medical care ($\phi _{1}$) increases after retirement and the health level without care ($\phi _{0}$) decreases then these factors can also imply higher demand. In the empirical analysis I address these possibilities by analysing how institutional characteristics, health, indicators of time constraints and health preferences influence the estimated effect of retirement on outpatient care use.

On the other hand, there is another mechanism in the model that implies decreasing health care demand after retirement. While working, income depends on health (through $W_{1}$), thus also on health care use. The positive effect of $W_{1}$ on *M* is reflected in the second part of Eq. 5. This financial incentive to be healthy disappears after retirement.^2^ This implication of the model is in line with the model solutions of Case and Deaton (2005) and Mazzonna and Peracchi (2012). To what extent this mechanism reduces the positive effect of retirement on health care use is an empirical question. The results of “Main results” section suggest that the positive effects dominate.

## Data

The main source of data I use in the paper is the Survey of Health, Aging and Retirement in Europe (SHARE).^3^ As part of the extensions, I compare in “Evidence from the US” section the results from Europe to the results based on the Health and Retirement Study (HRS)^4^ from the US. The SHARE is a multidisciplinary and cross-national panel database covering individuals aged 50 and above and their spouses. The survey runs bi-annually, the first wave of the data covers years 2004/2005. I use the first four waves of SHARE except for wave 3 data, which are retrospective (called SHARELIFE). I do not use data from wave 5 because GP and specialist visits are not differentiated in the survey of that wave. Although this is a panel database, there is variation in the country coverage across waves. I exclude Ireland and Israel from the empirical analysis because the income variable I use is not available for these two countries, Israel is an outlier in terms of the fieldwork times and of geographical location, and Ireland participated only in wave 2 of the survey.

I analyse the usage of outpatient care services, measured with four indicators: whether the respondent visited a GP or a specialist during the last twelve months, and the number of these visits. Due to item non-response, the number of observations vary across these four measures. I focus on outpatient care because individual preferences and resources are likely to have a stronger influence than on inpatient care use, which is mainly determined by health itself.

In most of the countries, GPs have some kind of gatekeeping role, i.e. a patient first has to see her GP if she wishes to contact a specialist. Also, countries commonly require some co-payments for some of the outpatient services. These institutional characteristics are summarised in Table 1.

**Table 1:** Gatekeeping and copayments in the analysed countries

	GP gatekeeper	GP not gatekeeper
Co-payments required	US, NL, SI, IT (specialist), DK (specialist), EE (specialist), FR (pensioners exempt), PT (social security beneficiaries exempt)	AT, SE, CH, CZ, DE (with incentives for gatekeeping), GR (pensioners exempt), BE (with incentives for gatekeeping; pre 2007: lower co-payments for pensioners)
No co-payments	ES, PL, IT(GP), DK(GP), EE(GP)	HU

*Source* Albreht et al. (2009), Anell et al. (2012), Barros et al. (2011), Bryndová et al. (2009), Busse and Blümel (2014), Chevreul et al. (2010), Economou (2010), European Observatory on Health Care Systems (2000), Ferré et al. (2014), Gaál et al. (2011), García-Armesto et al. (2010), Gerkens and Merkur (2010), Hofmarcher and Quentin (2013), Lai et al. (2013), Olejaz et al. (2012), Rice et al. (2013), Sagan et al. (2011), and Schäfer et al. (2010)

I present some descriptive statistics in Table 2. These statistics refer to the pooled sample. The sample is restricted to those individuals for whom the individual characteristics used in the analysis are non-missing (apart from health care use). This sample restriction facilitates the comparison of the results across the various specifications. The observations are weighted such a way that within each wave, each country has overall the same weight. Retirement is defined based on the self reported current job situation.

**Table 2:** Descriptive statistics, weighted SHARE data (pooled)

Variable	Mean	SD	Obs	Variable	Mean	Obs
GP, binary	0.813	0.390	111,779	Employment		
GP, count	4.443	7.080	111,779	Employee, not public	0.443	112,542
Specialist, binary	0.424	0.494	112,332	Employee, public	0.194	112,542
Specialist, count	1.853	5.105	106,106	Civil servant	0.107	112,542
Retired	0.538	0.499	112,456	Self-employed	0.136	112,542
Age	65.390	10.319	112,542	Other or not known	0.121	112,542
Female	0.559	0.496	112,542	Living area		
Income (EUR)	14,014	18,175	112,542	Big city	0.146	112,542
Illness, count	1.627	1.504	112,542	Suburbs big city	0.135	112,542
ADL limitations, count	0.229	0.826	112,542	Large town	0.176	112,542
Symptoms, count	1.811	1.917	112,542	Small town	0.231	112,542
Current smoker	0.190	0.393	112,542	Rural	0.313	112,542
Marital status				Subjective health		
Married	0.707		112,542	Excellent (very good)	0.092	112,542
Registered partnership	0.017		112,542	Very good (good)	0.202	112,542
Married, separated	0.011		112,542	Good (fair)	0.351	112,542
Never married	0.052		112,542	Fair (bad)	0.250	112,542
Divorced	0.071		112,542	Poor (very bad)	0.104	112,542
Widowed	0.143		112,542			
International standard classification of education (ISCED)						
Pre-primary	0.035		112,542			
Primary	0.233		112,542			
Lower secondary	0.181		112,542			
Secondary	0.320		112,542			
Post-secondary, not tertiary	0.038		112,542			
1st stage of tertiary	0.189		112,542			
2nd stage of tertiary	0.005		112,542			

Income is the average of the imputed annual gross household income values provided in SHARE, ppp adjusted, divided by household size. The indicators of illness (max 12), ADL limitations (max 6) and symptoms (max 13) are based on lists of possible conditions. The employment indicator refers to the current job, if exists, otherwise to the last job. For 14,000 observations the subjective health categories are very good/good/fair/bad/very bad

**Table 3:** Estimated average marginal effects, basic specifications, SHARE data

	(1)				(2)				(3)			
	GP, binary	Specialist, binary	GP, count	Specialist, count	GP, binary	Specialist, binary	GP, count	Specialist, count	GP, binary	Specialist, binary	GP, count	Specialist, count
Retired	0.0773$$^{***}$$	0.0382$$^{***}$$	1.156$$^{***}$$	0.241$$^{***}$$	0.0235$$^{***}$$	0.0213$$^{***}$$	$$-$$0.0448	0.149$$^{***}$$	0.0217$$^{***}$$	0.0218$$^{***}$$	0.148$$^{**}$$	0.185$$^{***}$$
	(0.00275)	(0.00345)	(0.0514)	(0.0389)	(0.00347)	(0.00439)	(0.0665)	(0.0510)	(0.00344)	(0.00432)	(0.0639)	(0.0512)
Age					0.0109$$^{***}$$	0.0194$$^{***}$$	0.165$$^{***}$$	0.108$$^{***}$$	0.00561$$^{***}$$	0.0118$$^{***}$$	0.100$$^{***}$$	0.0694$$^{**}$$
					(0.00148)	(0.00191)	(0.0278)	(0.0234)	(0.00142)	(0.00187)	(0.0263)	(0.0222)
Age squared					$$-$$5.20e−05$$^{***}$$	$$-$$0.000139$$^{***}$$	$$-$$0.000514$$^{***}$$	$$-$$0.000769$$^{***}$$	$$-$$3.28e−05$$^{***}$$	$$-$$0.000102$$^{***}$$	$$-$$0.000511$$^{***}$$	$$-$$0.000673$$^{***}$$
					(1.09e−05)	(1.39e−05)	(0.000197)	(0.000169)	(1.05e−05)	(1.36e−05)	(0.000187)	(0.000162)
Wave dummies	Yes	Yes	Yes	Yes	Yes	Yes	Yes	Yes	Yes	Yes	Yes	Yes
Country dummies	Yes	Yes	Yes	Yes	Yes	Yes	Yes	Yes	Yes	Yes	Yes	Yes
Individual controls	No	No	No	No	No	No	No	No	Yes	Yes	Yes	Yes
Observations	111,742	112,293	111,742	106,069	111,742	112,293	111,742	106,069	111,742	112,293	111,742	106,069

The individual controls are: gender, marital status, ISCED level, employment category, log income, living area, subjective health, and the number of illness, ADL limitations and symptoms

Clustered standard errors in brackets, $$^{***}$$ $$\hbox {p }{<}\,0.01$$, $$^{**}$$ $$\hbox {p}\,{<}\,0.05$$, $$^{*} \hbox { p}\,{<}\,0.1$$

## Empirical results

### Main results

#### Baseline results

Using the pooled SHARE data, I estimate binary outcome models with probit, and count data models with negative binomial regression.^5^ In the following tables I present the average marginal effects of the variables of main interest. The standard errors are clustered on the household level.

**Fig. 1:**
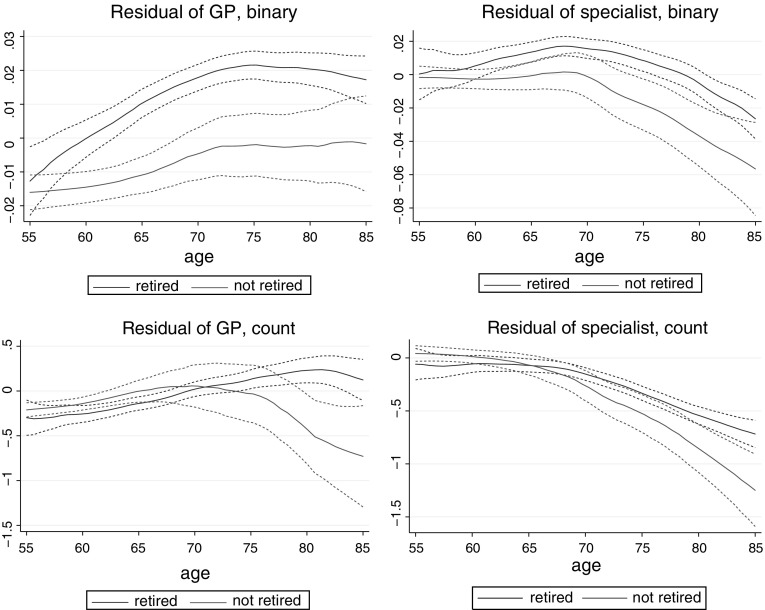
Effect of retirement on health care use (controlling for individual characteristics and wave dummies), local polynomial smooth with 95 % confidence interval, SHARE data

The first set of results are displayed in Table 3. In specification (1) I include wave and country dummies only. The results show that on average, retirees use more outpatient care than the rest of the 50+ population in Europe. Compared to the average number of visits, retirees have on average 26 % more GP visits and 13 % more specialist visits per year. These differences are statistically significant, and stronger for GP care than for specialist care. As specification (2) reveals, the higher use of outpatient care is partly explained by the age differences, as retirees are on average 15 years older than the rest of the population in the estimation sample. Including additional individual level control variables (specification (3)) has little effect on the estimated differences by retirement, except for the effect on the number of GP visits. Based on this specification, retirees are around 2 % points more likely to visit an outpatient physician, and the annual number of GP and specialist visits is on average 0.14–0.18 higher. This implies a 3–10 % higher number of visits than the average. Restricting the estimation sample to nonzero outcomes reveals that the positive estimated effects on the count outcomes as reported in Table 3, specification (3), are mainly driven by the increasing effect of retirement on the likelihood of outpatient care use, rather than by a positive effect on nonzero visits. If only the health controls are excluded from specification (3) then the estimated effect of retirement on health care use is slightly higher,^6^ indicating that without health controls the retirement effects would be overestimated.

To analyse the dynamics of how retirement influences outpatient care use, I plot the residuals of health care use (controlling for wave dummies and the individual controls used in specification (3), including health indicators, apart from retirement and age) as function of age, by retirement status. The plots of Fig. 1 indicate that the increasing effect of retirement on outpatient care use tends to appear at older ages. The marginal effect of retirement increases with age for all four dependent variables, which is the clearest for the binary indicator of GP visits. Since the typical age of retirement is around 65, the plots suggest that outpatient care use among the retirees is higher even long time after retirement.

#### Instrumental variables

Although retirement status might still be endogenous in specification (3) due to unobserved health problems linked both to retirement and health care use, the small difference between the results of specifications (2) and (3) and the increasing estimated retirement effect on the number of GP visits suggest that there are no such important endogeneity issues.

I apply two different instrumenting strategies to check the importance of endogeneity issues. First, I use data from MISSOC (2016) to generate gender and country specific indicators of early and full statutory retirement age, referring to year 2004, 2006 or 2010 (corresponding to survey wave 1, 2 and 4, respectively). This instrumenting strategy is standard in the literature, as also applied by Coe and Zamarro (2011) and Mazzonna and Peracchi (2012), among others. The statutory retirement ages can be assumed exogenous determinants of retirement, however, the instruments are subject to measurement error since in almost all of the analysed countries the official retirement ages are not homogeneous but depend among others on the years of service, service type, year of birth, or the number of children. Based on these indicators I generate two binary instrumental variables of whether someone is aged above the early and full retirement ages. Measurement errors in these variables weaken their strength as instruments and invalidate the zero conditional mean assumption of the vector of error terms in the estimated bivariate probit models, implying that the models are misspecified.^7^ Because of limitations of the MISSOC (2016) data, I follow a second instrumenting strategy, as well. Based on the SHARE data, I generate age, gender and country specific ratios of retired individuals as instrument for being retired.^8^ The drawback of this specification is that these ratios reflect individual retirement decisions, again implying endogeneity issues. I re-estimate specification (3) using these two alternative sets of instruments.

First, I re-estimate the binary outcome (probit) models with bivariate probit models, treating retirement as an endogenous variable, and using either the indicators of being above the statutory retirement age or the measure of retirement ratios as identifying variables for the effect of retirement on health care use. These indicators are strong predictors of retirement (Table 4).^9^ Identification of the bivariate probit model also comes from the functional form (assumption of joint normality of the error terms).

**Table 4:** Average marginal effects of the identifying variables on retirement, bivariate probit models, SHARE data

	(1)	(2)
	Retired	Retired
Above early ret age	0.101$$^{***}$$	
	(0.00435)	
Above full ret age	0.129$$^{***}$$	
	(0.00427)	
Mean retirement rate		0.573$$^{***}$$
		(0.00532)
Individual controls	Yes	Yes
Wave dummies	Yes	Yes
Country dummies	Yes	Yes
Observations	112,293	112,293

The individual controls are: age, age squared, gender, marital status, ISCED level, employment category, log income, living area, subjective health, and the number of illness, ADL limitations and symptoms

Heteroskedasticity robust standard errors in brackets, $$^{***} \hbox { p } {<}\,0.01$$, $$^{**} \hbox { p }{<}\,0.05$$, $$^{*} \hbox { p }{<}\,0.1$$

The estimated average marginal effects on the binary indicators of health care use are presented in the first part of Table 5. These suggest that if anything, the reported baseline results underestimate the true effects of retirement, possibly due to the unobserved health-preserving effect of retirement.

Second, I re-estimate the count data models using a generalised method of moments estimator of Poisson regression (using the “ivpois” Stata command of Nichols (2007)). The estimated marginal effects are reported in the second part of Table  5. These estimates are based on unweighted data without clustering (due to the limitations of the “ivpois” method), but the differences from the results reported in Table  3 are driven by the usage of the instrument. These results indicate that if exogenous retirement is assumed, the estimated effect of retirement on the number of GP visits might be slightly overestimated, whereas the effect on the number of specialist visits is underestimated.^10^ The magnitude of the over- and underestimation is relatively small.

**Table 5:** Marginal effect of retirement on outpatient care use with instrumental variables based on SHARE data

	GP, binary		Specialist, binary		GP, count		Specialist, count	
	(1)	(2)	(1)	(2)	(1)	(2)	(1)	(2)
Retired	0.0348$$^{***}$$	0.0303$$^{***}$$	0.0387$$^{***}$$	0.0511$$^{***}$$	0.121$$^{***}$$	0.0697$$^{***}$$	0.180$$^{**}$$	0.268$$^{***}$$
	(0.00787)	(0.00625)	(0.0110)	(0.00842)	(0.0364)	(0.0261)	(0.0783)	(0.0520)
Individual controls	Yes	Yes	Yes	Yes	Yes	Yes	Yes	Yes
Wave dummies	Yes	Yes	Yes	Yes	Yes	Yes	Yes	Yes
Country dummies	Yes	Yes	Yes	Yes	Yes	Yes	Yes	Yes
Observations	111,742	111,742	112,293	112,293	111,742	111,742	106,069	106,069

The binary outcome models are estimated with bivariate probit, the count data models with generalised method of moments estimator.

Instruments in specifications (1): being above the official early or full retirement age. In (2): age, gender, country specific retirement rate.

The individual controls are: age, age squared, gender, marital status, ISCED level, employment category, log income, living area, subjective health, and the number of illness, ADL limitations and symptoms

Standard errors in brackets (binary outcomes: clustered), $$^{***} \hbox { p } {<}\,0.01$$, $$^{**} \hbox { p } {<}\,0.05$$, $$^{*} \hbox { p } {<}\,0.1$$

The results suggest that treating the retirement indicator as exogenous lead to similar conclusions as if applying instrumental variables. In the following analysis I treat retirement as exogenous because of the difficulties related to instrumenting in the applied non-linear models. Even if there were endogeneity issues, the next set of estimations in which I investigate the driving factors of the positive relations between retirement and outpatient care use would remain valid as the results can reveal some underlying mechanisms of the correlations between retirement and outpatient care use.

#### Heterogeneity of retirement effects

My aim under the next set of specifications (Table 6) is to reveal which subgroups of the retired population drive the positive estimated effects on outpatient care use. This analysis also allows me to check two implications of the model of “Model” section, namely that changing time costs and health preferences can imply increasing health care use after retirement. First, in specification (4) I include an interaction term between being retired and the number of symptoms, as a representative indicator of health.^11^ The results indicate that retirement implies more outpatient care use among those who are in good health, which difference diminishes with the number of symptoms a respondent reports. Thus the relatively healthy seem more likely to increase outpatient care use after retirement. An explanation for this finding can be that individual suffering from severe health conditions visit the physicians irrespective of their employment status. However, those who have only minor illnesses might postpone such visits after retirement when they are less time constrained or pay more attention to minor health problems.

In specification (5) I interact the retirement indicator with the type of employment at the current or last job. Here I merge civil servant status with public employment. The biggest effect of retirement on outpatient care use is found among those who were self-employed at their last job. This holds for all four outcome variables, with the strongest difference for the number of GP visits. An explanation can be that self-employed people are more time-constrained than the rest of the working population. Based on the SHARE data, self-employed work on average longer hours than the not self-employed, the difference in the average is 2.5 h per week. Retirement relaxes this time constraint, implying a higher effect on health care use among the self-employed. This explanation is supported by the employment category marginal effects (not presented), where self-employment has a negative marginal effect on outpatient care use. Other possible explanations could be that the self-employed are more likely to retire due to health reasons or their health insurance status changes with retirement, however, these hypotheses are not supported by the data, using the reported reason of retirement and reported changes in health insurance coverage.

**Table 6:** Estimated average marginal effects, models extended with interaction terms, SHARE data

	(4)				(5)				(6)			
	GP, binary	Specialist, binary	GP, count	Specialist, count	GP, binary	Specialist, binary	GP, count	Specialist, count	GP, binary	Specialist, binary	GP, count	Specialist, count
Retired	0.0338$$^{***}$$	0.0415$$^{***}$$	0.604$$^{***}$$	0.477$$^{***}$$					0.0240$$^{***}$$	0.0245$$^{***}$$	0.146$$^{**}$$	0.201$$^{***}$$
	(0.00421)	(0.00533)	(0.0836)	(0.0691)					(0.00373)	(0.00458)	(0.0675)	(0.0548)
Retired $$\times $$ symptoms	$$-$$0.0091$$^{***}$$	$$-$$0.0106$$^{***}$$	$$-$$0.232$$^{***}$$	$$-$$0.149$$^{***}$$								
	(0.00182)	(0.00178)	(0.0229)	(0.0203)								
Retired $$\times $$ employee, not public					0.0188$$^{***}$$	0.0116$$^{*}$$	0.156$$^{**}$$	0.117				
					(0.00455)	(0.00564)	(0.0753)	(0.0715)				
Retired $$\times $$ employee, public					0.0109$$^{**}$$	0.0241$$^{***}$$	$$-$$0.0189	0.215$$^{***}$$				
					(0.00543)	(0.00691)	(0.103)	(0.0796)				
Retired $$\times $$ self-employed					0.0463$$^{***}$$	0.0345$$^{***}$$	0.607$$^{***}$$	0.340$$^{***}$$				
					(0.00726)	(0.00942)	(0.144)	(0.113)				
Retired $$\times $$ other					0.260$$^{***}$$	0.0340$$^{***}$$	0.0241	0.199$$^{*}$$				
					(0.00863)	(0.0103)	(0.139)	(0.104)				
Retired $\times$ smoke									$-$0.0101$^{*}$	$-$0.0139$^{*}$	0.0106	$-$0.0579
									(0.00613)	(0.00821)	(0.115)	(0.0984)
Smoke									$$-$$0.0473$$^{***}$$	$$-$$0.0472$$^{***}$$	$$-$$0.530$$^{***}$$	$$-$$0.363$$^{***}$$
									(0.00401)	(0.00558)	(0.0801)	(0.0716)
Wave dummies	Yes	Yes	Yes	Yes	Yes	Yes	Yes	Yes	Yes	Yes	Yes	Yes
Country dummies	Yes	Yes	Yes	Yes	Yes	Yes	Yes	Yes	Yes	Yes	Yes	Yes
Individual controls	Yes	Yes	Yes	Yes	Yes	Yes	Yes	Yes	Yes	Yes	Yes	Yes
Observations	111,742	112,293	111,742	106,069	111,742	112,293	111,742	106,069	111,742	112,293	111,742	106,069

Clustered standard errors in brackets, $$^{***} \hbox { p } {<}\,0.01$$, $$^{**} \hbox { p }{<}\,0.05$$, $$^{*} \hbox { p }{<}\,0.1$$. The individual controls are: age, age squared, gender, marital status, ISCED level, employment category, log income, living area, subjective health, and the number of illness, ADL limitations and symptoms

Next, I analyse whether health preferences can help explain the higher use of outpatient care among the retirees. I use smoking as a proxy for health preferences. The smoking indicator equals one if a respondent reports smoking at the present time ($19\,\%$ of the respondents in the estimation sample), zero otherwise. Specification (6) in Table 6 shows that on average, smokers are less likely to visit an outpatient physician, and their average number of outpatient visits is also smaller. However, controlling for smoking and its interaction with retirement has little effect on the estimated effect of retirement among the non-smokers. Although the results suggest that smoking implies a weaker effect of retirement (except for the effect on the number of GP visits), this weakening effect is only weakly significant statistically. If smoking captures health preferences then there is only weak evidence that the positive effect of retirement on outpatient care use were driven by individuals who are more health conscious, thus for whom health is more important.

There are at least two important problems with using smoking as a proxy for health preferences in the current application. Smoking can be considered as an addictive habit, therefore even if health preferences change after or around retirement, this might not be reflected in the smoking habits. On the one hand, this restricts to what extent we can observe changes in health preferences, on the other hand, this allows the results to be interpreted as the effect of retirement by long-run health preferences (as captured by smoking). Also, there is an endogeneity issue as more frequent physician visits might decrease the likelihood of smoking if the physician informs the patient on the adverse effects of smoking.

Comparing the empirical findings to the theoretical model of “Model” section indicates that the increasing effect of retirement on outpatient care use outweighs the potential decreasing effect due to the lower market incentives for maintaining good health. The empirical results are in line with being less time constrained and more health conscious after retirement.

### Cross country comparisons

#### Country by country analysis

In the previous section I estimated the effect of retirement on outpatient care use based on the pooled sample of SHARE. Country by country analysis can reveal if institutional differences as summarised in Table 1 have strong influence on the analysed relation. To some extent, I can also address the effect of prices on the relations between retirement and outpatient care use. The scope for country by country analysis is limited by the small country specific sizes of the sample.

The estimated country specific marginal effects of retirement are presented in Table 7. For all countries and for all four dependent variables a significantly positive or insignificant (but generally still positive) effect is estimated, except for the puzzling negative marginal effect of being retired on the number of specialist care visits in Poland and the probability of GP visits in Estonia. Another finding is that the estimated effects tend to be larger in the Southern countries, namely Greece, Italy and Spain. Although indicators of health are controlled for, this cross country difference might still partly reflect that in the Southern countries individuals select more into retirement based on health care needs. The estimated effects are generally less precise for countries with fewer total number of observations, in particular for countries for which only one wave of data are available (Hungary, Portugal, Slovenia, Estonia).

**Table 7:** Estimated average marginal effect of retirement in country specific models of outpatient care use

	GP, binary	Specialist, binary	GP, count	Specialist, count
Austria	0.0144	0.0369$$^{**}$$	0.468$$^{***}$$	0.140
	(0.0118)	(0.0153)	(0.171)	(0.143)
Germany	0.0117	0.0309$^{*}$	0.345	0.0531
	(0.0123)	(0.0175)	(0.210)	(0.199)
Sweden	0.0459$$^{***}$$	0.00157	0.134	0.319$$^{***}$$
	(0.0170)	(0.0177)	(0.0999)	(0.0969)
Netherlands	0.0308$$^{**}$$	0.0325$$^{**}$$	$$-$$0.0229	0.398$$^{***}$$
	(0.0133)	(0.0143)	(0.0918)	(0.154)
Spain	0.0308$$^{***}$$	0.0438$$^{***}$$	0.582$$^{**}$$	0.520$$^{***}$$
	(0.0115)	(0.0150)	(0.280)	(0.155)
Italy	0.0263$$^{**}$$	0.0498$$^{***}$$	0.291	0.181
	(0.0107)	(0.0140)	(0.287)	(0.142)
France	0.00893	0.0173	0.112	0.115
	(0.00799)	(0.0135)	(0.129)	(0.122)
Denmark	$-$0.0193	$-$0.0244	0.113	$-$0.237
	(0.0165)	(0.0179)	(0.167)	(0.174)
Greece	0.0126	0.0580$$^{***}$$	0.304	0.499$$^{***}$$
	(0.0181)	(0.0180)	(0.207)	(0.175)
Switzerland	0.0458$$^{***}$$	0.00998	0.146	0.251
	(0.0162)	(0.0186)	(0.152)	(0.189)
Belgium	0.0271$$^{***}$$	0.0220$$^{*}$$	0.185	0.0361
	(0.00824)	(0.0126)	(0.164)	(0.117)
Czech Rep.	0.0105	0.0327$$^{*}$$	0.444$$^{**}$$	0.115
	(0.0121)	(0.0175)	(0.201)	(0.247)
Poland	0.0397$$^{**}$$ $$^{**}$$	$$-$$0.0337	0.280	$$-$$0.677$$^{***}$$
	(0.0182)	(0.0211)	(0.293)	(0.230)
Hungary	0.0311	0.0487$^{*}$	0.283	0.394
	(0.0198)	(0.0260)	(0.377)	(0.314)
Portugal	0.0146	$-$0.0402	$-$0.646	$-$0.260
	(0.0255)	(0.0304)	(0.457)	(0.310)
Slovenia	0.0242	$-$0.00942	0.0780	0.173
	(0.0265)	(0.0259)	(0.362)	(0.202)
Estonia	$$-$$0.0307$$^{**}$$	$$-$$0.0126	$$-$$0.234	0.106
	(0.0153)	(0.0163)	(0.243)	(0.155)

The included control variables are: wave dummies, age, age squared, gender, marital status, ISCED level, employment category, log income, living area, subjective health, and the number of illness, ADL limitations and symptoms

Clustered standard errors in brackets, $$^{***} \hbox { p } {<}\,0.01$$, $$^{**} \hbox { p }{<}\,0.05$$, $$^{*} \hbox {p}\,{<}\,0.1$$

#### Institutional differences

The cross country coverage of the SHARE data allows me to analyse to what extent the effect of retirement on outpatient care use differs according to the institutional differences across the countries. In all the specifications of this subsection I re-estimate specification (3) of Table 3 with the only difference that the estimation samples are selected based on certain country-specific institutional characteristics. In this way I allow the effect of all the regressors to differ between the country groups. The results are summarised in Table 8.

First, I analyse the heterogeneity with respect to the gatekeeper role of GPs. The results indicate that the gatekeeping role of GPs decreases the estimated effect of retirement not only on specialist care use but also on GP care use.

Next, I investigate if co-payments have an influence on the effect of retirement on outpatient care use. The results suggest that the positive effect of retirement on the expected number of GP visits is smaller in countries where co-payments are required for GP care visits. There is no evidence that the retirement effect on specialist care would diminish with the existence of co-payment requirements.

Finally, I check if the estimated effect of retirement on outpatient care use is bigger in countries where pensioners are exempt from co-payments or have to pay reduced fees. According to Table 1, this applies to Belgium, France, Greece and Portugal. I do not find evidence for such differences. This simple analysis suggests that price changes (decreasing *p* in the model of “Model” section) cannot explain the observed increase in outpatient care use after retirement.

**Table 8:** Effect of retirement on outpatient care use by institutional characteristics, SHARE data

	GP, binary		Specialist, binary		GP, count		Specialist, count	
Gatekeeping	No	Yes	No	Yes	No	Yes	No	Yes
Retired	0.0256$$^{***}$$	0.0189$$^{***}$$	0.0297$$^{***}$$	0.0143$$^{**}$$	0.332$$^{***}$$	$$-0.0283$$	0.290$$^{***}$$	0.0862
	(0.00502)	(0.00474)	(0.00635)	(0.00589)	(0.0805)	(0.0964)	(0.0769)	(0.0657)
Observations	55,712	56,030	56,002	56,291	55,712	56,030	52,108	53,961
GP co-payments	No	Yes			No	Yes		
Retired	0.0198$$^{***}$$	0.0238$$^{***}$$			0.210$$^{*}$$	0.136$$^{*}$$		
	(0.00589)	(0.00426)			(0.113)	(0.0752)		
Observations	35,673	76,069			35,673	76,069		
Specialist co-payments			No	Yes			No	Yes
Retired			0.0234$$^{**}$$	0.0219$$^{***}$$			0.142	0.195$$^{***}$$
			(0.0112)	(0.00468)			(0.117)	(0.0561)
Observations			14,430	97,863			13,853	92,216
Co-payments decrease with retirement	No	Yes	No	Yes	No	Yes	No	Yes
Retired	0.0227$$^{***}$$	0.0179$$^{***}$$	0.0224$$^{***}$$	0.0223$$^{***}$$	0.216$$^{***}$$	$$-0.0763$$	0.210$$^{***}$$	0.101
	(0.00407)	(0.00608)	(0.00501)	(0.00822)	(0.0662)	(0.151)	(0.0608)	(0.0903)
Observations	81,348	30,394	81,770	30,523	81,348	30,394	77,223	28,846

The included control variables are: wave dummies, age, age squared, gender, marital status, ISCED level, employment category, log income, living area, subjective health, and the number of illness, ADL limitations and symptoms

Clustered standard errors in brackets, $$^{***} \hbox { p}\,<\,0.01$$, $$^{**} \hbox { p}\,<0.05$$, $$^{*} \hbox { p}\,<0.1$$

#### Evidence from the US

How is retirement related to outpatient physician visits in the US, in comparison to Europe? The effect of retirement on outpatient care use in the US is analysed based on data from the HRS. This data set served as a sample for the SHARE data, thus the structure and the topics covered are similar. I use data from the first 11 waves of the HRS, covering years 1992–2012. In the HRS there is no separate information on the usage of GP and specialist care facilities. I estimate models of the binary indicator of outpatient physician visit and of the count of these visits. There are some differences across waves how the outpatient care use is measured. The reference year is the last year in wave 1, whereas the last two years in the other waves. In all waves the respondents are instructed not to include hospital stays in the reported number of doctoral visits, but in waves 1 and 2 they are also instructed not to include nursing home stays. Because of the different length of the reference period I omit wave 1 observations from the estimations. The other discrepancies across waves are handled by including wave dummies in all specifications. The binary variable “retired” equals one only for the full time retired individuals.

Specification (1) is similar to the third specification of Table 3. I estimate probit and negative binomial models of the two outcome indicators, including individual characteristics and wave dummies as regressors. Specification (2) is a first differenced (FD) model (which gives similar results to a fixed effects specification).

**Table 9:** Estimated average marginal effect (1) and coefficients (2) of retirement on outpatient doctoral visits based on the HRS data

	(1)		(2)	
	Doctoral visits, binary	Doctoral visits, count	FD binary	FD count
Retired	0.00459$$^{**}$$	0.729$$^{***}$$	$$-0.00219$$	0.533$$^{*}$$
	(0.00205)	(0.133)	(0.00306)	(0.307)
Age	0.00198$$^{**}$$	$$-$$0.0903		
	(0.000899)	(0.0654)		
Age squared	$$-$$1.38e−05$$^{**}$$	0.000393	1.33e−05	0.00496$$^{***}$$
	(6.85e−06)	(0.000489)	(1.26e−05)	(0.000971)
Wave dummies	Yes	Yes	Yes	Yes
Individual controls	Yes	Yes	Yes	Yes
Observations	129,429	126,246	105,030	100,455

Individual controls in specification (1): gender, race, 5-level education, marital status, industry of job with longest tenure, Census Division of residence, indicators of health insurance status, self reported health, number of ever diagnosed health conditions (0 to 6) and ADL limitations. Individual controls in specification (2): widowhood, indicators of health insurance status, self reported health, number of health conditions ever diagnosed and ADL limitations

Clustered standard errors in brackets ((2): robust), $$^{***} \hbox { p }{<}\,0.01$$, $$^{**} \hbox { p }{<}\,0.05$$, $$^{*}\hbox { p }{<}\,0.1$$

A robust finding is that retirement implies higher number of outpatient doctoral visits in the US, ceteris paribus. This finding is in contrast to the results of the earlier literature, as discussed in “Introduction” section. The estimated effect is somewhat larger than based on the European sample, however, here the effects refer to 2-year utilisation. The estimated effects on the number of visits are qualitatively robust to the estimation method, although quantitatively the effects decrease with first differencing. With a mean number of visits of around 10, the estimated effects are moderate, comparable in relative terms to the European estimates. There is no clear evidence that retirement would imply a higher probability of outpatient doctoral visits. Since the average likelihood of reporting an outpatient visit is above 90 %, the small estimated effects are not surprising.

To check whether unobserved health or other effects drive the estimation results, I apply a similar method as in “Instrumental variables” section for the SHARE data. I re-estimate the probit and count data models and the first differenced models with using the binary indicators of being aged above 62 and 65 as instrument for retirement.^12^ Similar instrumenting strategy is applied by Bonsang et al. (2012), among others, and the rationale behind is that the early (62) and full (65) retirement ages act as exogenous predictors of retirement. The results are reported in Table 10. Looking at the pooled estimates with instrumental variables, the results indicate that the magnitude of the effects of retirement on the probability and number of doctoral visits might be overestimated, although the positive effects are robust results. However, if the instruments are used in the first differenced models then the standard errors become relatively large, hence the point estimates are unreliable. A further limitation of this instrumenting strategy is that being aged above 65 also implies Medicare eligibility, implying direct effect of the instrument on health care use (as also pointed out by Eibich 2015).

**Table 10:** Estimated effect of retirement on outpatient doctoral visits using instrumental variables based on the HRS data

	Bivariate probit	GMM	2SLS, FD binary	2SLS, FD count
Retired	0.00376	0.183	0.00718	$-0.310$
	(0.0248)	(0.119)	(0.0330)	(2.248)
Individual controls	Yes	Yes	Yes	Yes
Wave dummies	Yes	Yes	Yes	Yes
Observations	129,429	126,246	105,030	100,455
First stage F-stat			274.932$$^{***}$$	267.975$$^{***}$$

Individual controls in the pooled models: gender, race, 5-level education, marital status, industry of job with longest tenure, Census Division of residence, indicators of health insurance status, self reported health, number of ever diagnosed health conditions (0–6) and ADL limitations

Individual controls in the FD models: widowhood, indicators of health insurance status, self reported health, number of health conditions ever diagnosed and ADL limitations

Clustered standard errors in brackets, $$^{***} \hbox {p }{<}\,0.01$$, $$^{**} \hbox { p }{<}\,0.05$$, $$^{*} \hbox { p } {<}\,0.1$$

**Fig. 2:**
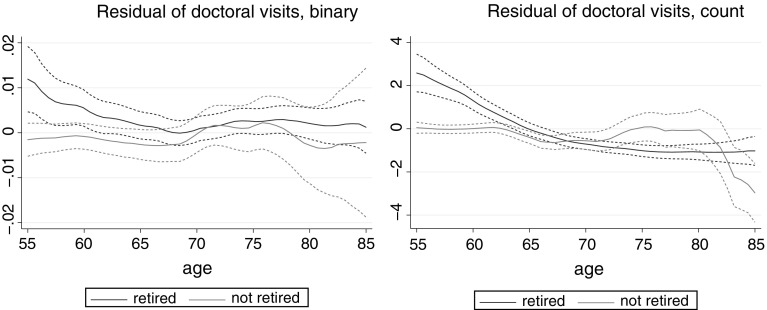
Effect of retirement on health care use (controlling for individual characteristics and wave dummies), local polynomial smooth with 95 % confidence interval, HRS data

Specification (2) of Table 9 indicates that retirement increases the number of outpatient visits in the short run (within two years). If the first lag of transition to retirement is included as a regressor instead of the current one then a significant negative effect is estimated. The positive short run effect is contrary to the SHARE results which indicate stronger long run effects of retirement on outpatient care use. This difference is also reflected in the plots of Fig. 2. The European data suggest increasing effect of retirement with age, whereas the US data suggest a decreasing effect both on the probability of outpatient care use and on the number of doctoral visits. An explanation for the short run effect in the US could be that insurance status changes with retirement, gaining coverage for doctoral visits. However, insurance coverage is controlled for, and changes in insurance status with retirement are more common at age 65 and above when Medicare Part B eligibility kicks in, while the figures indicate positive effects of retirement on outpatient care use at earlier ages. Unobserved insurance conditions can still contribute to the results. As the results with instrumental variables also suggest, unobserved health might also drive part of the estimated retirement effects, in particular at younger ages. The small or even negative long run effects of retirement in the US might also be due to the fact that most people still have to pay co-payments for doctoral visits, typically 20 % (which is the rate of co-payments under Medicare Part B). Also, in the US, there is less scope for retirement to increase the probability of having a doctoral visit at older ages. Based on the surveys used, 93 % of the not retired respondents aged 65 and above report a doctoral visits, while the corresponding statistic for GP visits in Europe is 83 % (and 43 % for specialist visits). Thus, even without being retired, almost all respondents report a doctoral visit at older ages in the US, therefore retirement cannot have a substantial effect.

## Conclusions

Retirees use more outpatient services than the rest of the population of similar age, both in Europe and in the US. The utility maximisation model of health care demand derived in this paper suggests that there can be multiple channels which contribute to this positive gradient. Declining health after retirement, changing health preferences, decreasing opportunity cost of time and institutional settings can all lead to the observed positive relation. The empirical analysis sheds light on whether these possible mechanisms indeed play a role. Such an in-depth analysis of the relation of retirement to outpatient care use is a novelty in the literature, as well as the analysis of the relation based on European data. The empirical analysis is based on the European SHARE data, and the results are compared to US results based on data from the HRS.

The results suggest that within Europe, a major part of the retirement—outpatient care gradient is explained by age, health status, and other individual characteristics. Controlling for these factors, the average number of GP and specialist visits per year is only 0.15–0.19 (3–10 %) higher among the retirees than among the rest of the 50+ sample. A very rough calculation (assuming 50$ cost of an outpatient visit in line with estimates of WHO (2016) and 3,300$ per capita health expenditures, which is close to the OECD average) indicates that it implies around 0.25 % increase in per capita health expenditures due to retirement. These results are qualitatively robust to using official retirement ages or population level retirement statistics as instruments for retirement. While these estimates suggest only a small financial burden of the additional outpatient care use due to retirement, in some European countries the effects are much larger, reaching around 1 % of the per capita spending on outpatient care. Further estimations reveal that the positive relations are mostly driven by the healthier individuals, by the ones who could be considered as more time-constrained while working, and by those who have stronger health preferences. Country-by-country analysis also reveals that the gatekeeping role of GPs decreases the effect of retirement on specialist care use and co-payments decrease the effect of retirement on GP visits. According to the estimation results, a major difference in the gradient between Europe and US is that while in the US the positive effect of retirement on outpatient care use can be observed right after retiring, there is no such evidence for a short-run effect in Europe. Also, unlike in Europe, no evidence is found for positive long-run effects of retirement on outpatient care use in the US. The insurance coverage structure and required co-payments in the US can provide explanation for these differences, however, data limitations have to be kept in mind when drawing further conclusions from this comparison.

An important policy relevant question is whether the higher use of outpatient care by the retirees is welfare improving, or it rather creates an unintended burden on the health care budget. The results of this paper can only provide some indicative answers to this question. Part of the retirement—outpatient care gradient can be accounted for by other observable characteristics, such as age and health. With ageing the need for health care increases, which coincides with retirement. This part of the increased demand can be considered as justified. Also, a part of the increased utilisation can in fact be due to better access to care. Before retirement, the access might be limited by time constraints (e.g. self-employed in Europe) or by money constraints (e.g. in the US or in countries with substantial co-payments in Europe). The removal of these constraints with retirement can in the end lead to health improvements and more equal access to outpatient care. Health improvements are especially likely if the increased health care use implies extended use of preventive services. Thus the positive estimated effect of retirement on outpatient care use does not necessarily contradict the findings of the literature on the positive health effects of retirement. At the same time, there seems to be a part of the health care use—retirement gradient which can be due to excess demand occurring after retirement. This is reflected by the estimated mitigating role of gatekeeping, and by the stronger retirement effect among the healthy individuals. Ideally, once health is controlled for, there should be no retirement effect on health care use. Any estimated effect is either due to time or money constraints before retirement, together with the lack of attention paid to one’s own health, or to excess demand after retirement.

## Appendix

I present here the detailed solution of the model of “Model” section. The maximisation problem with consumption (*C*), health (*H*), and health care use (*M*) is:

6 $$\begin{aligned} \max _{C\ge 0,M\ge 0}U= & {} C^{\gamma }H^{1-\gamma } \end{aligned}$$

7 $$\begin{aligned} H= & {} \phi _{0}\left( \rho \right) +\phi _{1}M;\, \phi _{0},\phi _{1}\ge 0. \end{aligned}$$

The budget constraint is:

8 $$\begin{aligned} C+pM=\rho R+(1-\rho )w\left( W_{0}+W_{1}H-tM\right) . \end{aligned}$$

The parameters are defined in “Model” section. The maximisation problem can be rewritten as:

9 $$\begin{aligned} \max _{M\ge 0}U= & {} \left( \rho R+(1-\rho )w\left[ W_{0}+W_{1}\left( \phi _{0}\left( \rho \right) +\phi _{1}M\right) -tM\right] -pM\right) ^{\gamma }\nonumber \\&\times \left( \phi _{0}\left( \rho \right) +\phi _{1}M\right) ^{1-\gamma }. \end{aligned}$$

The first order condition is:

10 $$\begin{aligned}&\gamma \left[ (1-\rho )w\left( W_{1}\phi _{1}-t\right) -p\right] \nonumber \\&\quad \times \left( \frac{\phi _{0}\left( \rho \right) +\phi _{1}M}{\rho R+(1-\rho )w\left[ W_{0}+W_{1}\phi _{0}\left( \rho \right) +\left( W_{1}\phi _{1}-t\right) M \right] -pM}\right) ^{1-\gamma }+\left( 1-\gamma \right) \phi _{1} \nonumber \\&\quad \times \left( \frac{\rho R+(1-\rho )w\left[ W_{0}+W_{1}\phi _{0}\left( \rho \right) +\left( W_{1}\phi _{1}-t\right) M \right] -pM}{\phi _{0}\left( \rho \right) +\phi _{1}M}\right) ^{\gamma }=0 \end{aligned}$$

This can be simplified to:

11 $$\begin{aligned}&\gamma \left[ (1-\rho )w\left( W_{1}\phi _{1}-t\right) -p\right] +\left( 1-\gamma \right) \phi _{1}\nonumber \\&\quad \times \frac{\rho R+(1-\rho )w\left[ W_{0}+W_{1}\phi _{0}\left( \rho \right) +\left( W_{1}\phi _{1}-t\right) M\right] -pM}{\phi _{0}\left( \rho \right) +\phi _{1}M}=0 \end{aligned}$$

After some rearrangements:

12 $$\begin{aligned}&\gamma \left[ (1-\rho )w\left( W_{1}\phi _{1}-t\right) -p\right] \left( \phi _{0}\left( \rho \right) +\phi _{1}M\right) \nonumber \\&\quad =\left( \gamma -1\right) \phi _{1}\left( \rho R+(1-\rho )w\left[ W_{0}+W_{1}\phi _{0}\left( \rho \right) +\left( W_{1}\phi _{1}-t\right) M \right] -pM\right) , \nonumber \\&M=-\frac{\gamma \left[ (1-\rho )w\left( W_{1}\phi _{1}-t\right) -p\right] \phi _{0}\left( \rho \right) +\left( 1-\gamma \right) \phi _{1}\left( \rho R+(1-\rho )w\left[ W_{0}+W_{1}\phi _{0}\left( \rho \right) \right] \right) }{ \left( 1-\gamma \right) \phi _{1}\left( (1-\rho )w\left( W_{1}\phi _{1}-t\right) -p\right) +\gamma \phi _{1}\left[ (1-\rho )w\left( W_{1}\phi _{1}-t\right) -p\right] }\nonumber \\&\quad \;\;=-\gamma \frac{\phi _{0}\left( \rho \right) }{\phi _{1}}-\frac{\left( 1-\gamma \right) \left( \rho R+(1-\rho )w\left[ W_{0}+W_{1}\phi _{0}\left( \rho \right) \right] \right) }{(1-\rho )w\left( W_{1}\phi _{1}-t\right) -p}. \end{aligned}$$

For the retired ($\rho =1$) the optimal demand for medical care therefore is:

13 $$\begin{aligned} M_{\rho =1}=-\gamma \frac{\phi _{0}\left( \rho =1\right) }{\phi _{1}}+\frac{ \left( 1-\gamma \right) R}{p}. \end{aligned}$$

For the working population ($\rho =0$) the optimal demand is:

14 $$\begin{aligned} M_{\rho =0}=-\gamma \frac{\phi _{0}\left( \rho =0\right) }{\phi _{1}}+\frac{ \left( 1-\gamma \right) w\left[ W_{0}+W_{1}\phi _{0}\left( \rho \right) \right] }{p-w\left( W_{1}\phi _{1}-t\right) }. \end{aligned}$$

The second order condition is satisfied. To show this, the second order condition after some simplifications is:

15 $$\begin{aligned}&\partial \left( \frac{\phi _{0}+\phi _{1}M}{\rho R+(1-\rho )w\left[ W_{0}+W_{1}\phi _{0}+\left( W_{1}\phi _{1}-t\right) M\right] -pM}\right) \Bigg /\partial M\nonumber \\&\quad \times \left\{ \left[ (1-\rho )w\left( W_{1}\phi _{1}-t\right) -p\right] \right. \nonumber \\&\quad \left. \times \left( \frac{\phi _{0}+\phi _{1}M}{\rho R+(1-\rho )w\left[ W_{0}+W_{1}\phi _{0}+\left( W_{1}\phi _{1}-t\right) M\right] -pM}\right) -\phi _{1}\right\} <0. \end{aligned}$$

To save notation, I did not indicate above that the $\phi _{0}$ parameter depends on the retirement status ($\rho$). For the retired this simplifies to:

16 $$\begin{aligned} \left( \frac{R}{p}+\frac{\phi _{0}\left( \rho =1\right) }{\phi _{1}}\right) \cdot \frac{p\phi _{0}\left( \rho =1\right) +\phi _{1}R}{R-pM}>0, \end{aligned}$$

implying that the monetary cost of medical care should be smaller than the retirement income, which is implied by the nonnegativity of consumption (*C*).

For the working population the second order condition is:

17 $$\begin{aligned}&\partial \left( \frac{\phi _{0}+\phi _{1}M}{w\left[ W_{0}+W_{1}\phi _{0}+\left( W_{1}\phi _{1}-t\right) M\right] -pM}\right) \Bigg /\partial M\nonumber \\&\quad \times \left\{ \left[ w\left( W_{1}\phi _{1}-t\right) -p\right] \left( \frac{\phi _{0}+\phi _{1}M}{w\left[ W_{0}+W_{1}\phi _{0}+\left( W_{1}\phi _{1}-t\right) M\right] -pM}\right) -\phi _{1}\right\} <0.\nonumber \\ \end{aligned}$$

Using that

18 $$\begin{aligned}&\partial \left( \frac{\phi _{0}+\phi _{1}M}{w\left[ W_{0}+W_{1}\phi _{0}+\left( W_{1}\phi _{1}-t\right) M\right] -pM}\right) \Bigg /\partial M \nonumber \\&=\frac{ \phi _{1}wW_{0}+\phi _{0}\left( wt+p\right) }{(w\left[ W_{0}+W_{1}\phi _{0}+\left( W_{1}\phi _{1}-t\right) M\right] -pM)^{2}}>0, \end{aligned}$$

the second order condition simplifies further to:

19 $$\begin{aligned} \left[ w\left( W_{1}\phi _{1}-t\right) -p\right] \left( \frac{\phi _{0}+\phi _{1}M}{w\left[ W_{0}+W_{1}\phi _{0}+\left( W_{1}\phi _{1}-t\right) M\right] -pM}\right) -\phi _{1}<0, \end{aligned}$$

which can be rearranged to (and indicating that $\phi _{0}$ is a function of $\rho$):

20 $$\begin{aligned} \frac{wt\phi _{0}\left( \rho =1\right) +p\phi _{0}\left( \rho =1\right) +\phi _{1}wW_{0}}{w\left[ W_{0}+W_{1}(\phi _{0}\left( \rho =1\right) +\phi _{1}M)-tM\right] -pM}>0. \end{aligned}$$

This is again implied by the nonnegativity of consumption (*C*).

## Footnotes

1 I assume here that someone is either working or retired.

2 I thank an anonymous referee for pointing out this additional mechanism.

3 This paper uses data from SHARE wave 4 release 1.1.1, as of March 28th 2013 (doi:10.6103/SHARE.w4.111) and SHARE wave 1 and 2 release 2.6.0, as of November 29 2013 (doi:10.6103/SHARE.w1.260 and 10.6103/SHARE.w2.260). The SHARE data collection has been primarily funded by the European Commission through the 5th Framework Programme (project QLK6-CT-2001-00360 in the thematic programme Quality of Life), through the 6th Framework Programme (projects SHARE-I3, RII-CT-2006-062193, COMPARE, CIT5- CT-2005-028857, and SHARELIFE, CIT4-CT-2006-028812) and through the 7th Framework Programme (SHARE-PREP, $\hbox {No.}$ 211909, SHARE-LEAP, $\hbox {No.}$ 227822 and SHARE M4, $\hbox {No.}$ 261982). Additional funding from the U.S. National Institute on Aging (U01 AG09740-13S2, P01 AG005842, P01 AG08291, P30 AG12815, R21 AG025169, Y1-AG-4553-01, IAG BSR06-11 and OGHA 04-064) and the German Ministry of Education and Research as well as from various national sources is gratefully acknowledged (see http://www.share-project.org for a full list of funding institutions).

4 I use the RAND HRS Data, Version N. This data set is produced by the RAND Center for the Study of Aging. The HRS is sponsored by the National Institute on Aging (Grant No. NIA U01AG009740) and is conducted by the University of Michigan. The RAND HRS Data file is an easy to use longitudinal data set based on the HRS data. It was developed at RAND with funding from the National Institute on Aging and the Social Security Administration.

5 The results are robust to restricting the sample to the first observation per each respondent, instead of using the pooled data.

6 Excluding health controls, the estimated marginal effect of retirement on GP binary, specialist binary, GP count, specialist count is 0.0280, 0.0268, 0.191, 0.194, respectively, all statistically significant at the 1 % level.

7 For a discussion of measurement errors in bivariate probit models, see Roy and Banerjee (2009).

8 In their working paper, Bonsang et al. (2010) use a similar instrumental variable for retirement in the SHARE data.

9 Instead of the bivariate probit models, the method of two-stage least squares (2SLS) could also be applied, although that does not take into account the binary nature of the outcome variables and the retirement indicator. For discussion of the bivariate probit models, see Angrist and Pischke (2008) and Chiburis et al. (2012). The 2SLS estimates of the effect of retirement on the binary outcomes are slightly above the bivariate probit results. First stage statistics indicate that the applied instruments are strong, as also suggested by the results of Table 4.

10 2SLS estimated coefficient of retirement is even larger and statistically significant, except for the effect on GP visits if mean retirement rate is used as instrument, in which case the retirement coefficient is insignificant.

11 The correlation coefficient of the number of symptoms with the number of illnesses is 0.59, with the number of ADL limitation is 0.36, and with the subjective health measure is 0.52.

12 As in “Instrumental variables” section, I estimate a bivariate probit model and a generalised method of moments (GMM) estimator of Poisson regression. When instrumenting the first differenced retirement, I estimate two stage least squares models.
